# Urinary incontinence in a fitness club setting—is it a workout problem?

**DOI:** 10.1007/s00192-020-04253-0

**Published:** 2020-03-04

**Authors:** Lene A. H. Haakstad, Christina Gjestvang, Tayla Lamerton, Kari Bø

**Affiliations:** 1 Norwegian School of Sports Sciences, Department of Sports Medicine, P.O. Box 4014, Ullevål Stadion, 0806 Oslo, Norway; 2 Human School of Sport and Nutrition Science, University of Queensland, Brisbane, Australia; 3 Department of Obstetrics and Gynecology, Akershus University Hospital, Lørenskog, Norway

**Keywords:** Fitness club, Exercise, Pelvic floor muscle training, Urinary incontinence, Women’s health

## Abstract

**Introduction:**

The aims of the present study were to report longitudinal data on the prevalence of urinary incontinence (UI) in a fitness club setting and to investigate whether gym members are educated about and exercise their pelvic floor muscles.

**Methods:**

New members (125 women) from 25 fitness clubs in Oslo, Norway, filled in a 25-min online questionnaire (SurveyXact) at four time points (onset, 3, 6 and 12 months of fitness club membership). The questionnaire covered background/health information, membership dropout and exercise habits, including pelvic floor muscle training (PFMT). A modified Subjective Health Complaints Inventory (SHC Inventory) and the International Consultation on Incontinence Questionnaire-Urinary Incontinence Short Form (ICIQ-UI SF) were used to gather repeated measures of UI.

**Results:**

At onset, 3, 6 and 12 months of fitness club membership, 16.8%, 13.8%, 19.6% and 18.7% reported UI, respectively (*p* = 0.11). Of these, 57.1% to 76.2% reported leakage during exercise and perceived the UI to be slight. Less than 8% had received information about PFMT by the fitness club staff. Adherence to regular exercise and PFMT throughout the follow-up period (minimum two sessions/week) did not show any association with absent or present UI at 12 months (*p* = 0.48 and *p* = 0.63) and was reported by 30% and 22.2% of the participants, respectively.

**Conclusions:**

About 17% reported UI at onset of fitness club membership, with no changes in proportions throughout the first year. Adherence to regular exercise and PFMT did not show any association with absent or present UI at 12 months. Few had been taught PFMT.

## Introduction

Urinary incontinence (UI) is defined as involuntary leakage of urine and is a common health complaint, affecting millions of women worldwide [1]. Prevalence rates vary greatly because of the heterogeneity of study populations and different classifications of UI, yet most research shows data in the range of 25–45% [2]. Leakage during physical activity, coughing, sneezing and laughing is classified as stress urinary incontinence (SUI), whereas leakage with the urge to void is classified as urgency urinary incontinence (UUI) [3, 4].

Pregnancy, vaginal delivery, high body mass index (BMI) and being middle-aged (≥ 40 years) are known risk factors for the development of UI [2, 5, 6]. However, studies have also shown that nulliparous young women experience UI, especially women participating in high-impact activities and sports [7, 8]. Exercise increases intra-abdominal pressure and the impact from ground reaction forces. Overload and fatigue of the pelvic floor muscles arising from repetitive contractions, combined with sudden changes in pressure during exercise, may increase the risk of leaking urine [9].

According to a systematic review, incontinence during exercise ranges from 14.9% to 80%, depending on the type of activity [8]. UI may be an important barrier to exercise, and women may change their activity pattern or drop out of regular exercise because of UI [8, 10]. However, some studies have found that low-impact activities (such as walking) decrease the incidence and prevalence of UI [8]. However, these studies are cross sectional and may be limited by selection bias, so it is difficult to conclude whether women do not leak because they exercise or whether they can exercise because they do not leak. It may also be that the relationship between low-impact activities and decreased risk of UI is mediated by prevention of weight gain, given that overweight and obesity are strong predictors of UI [6, 8].

As early as 1948, Kegel [11] suggested pelvic floor muscle training (PFMT) for the prevention and treatment of UI. Since then, many high-quality randomized controlled trials (RCTs) and meta-analyses have been conducted, and to date PFMT is recommended as first-line treatment for women with UI [12]. The best results are obtained for SUI with close follow-up and supervised training [12].

Much research has focused on UI in athletes, including group fitness instructors [7, 8, 13]. A few studies have also reported the prevalence of UI among regular exercisers, including young women who participate in high-impact and high-intensity exercise [14–16]. However, there is a lack of knowledge about beginner recreational exercisers in a fitness club setting. Hence, the main aim of the present study was to report the prevalence of UI in an age-diverse group of women across four time points: at onset, 3, 6 and 12 months of fitness club membership. In addition, we wanted to investigate whether gym members have awareness/knowledge about PFMT and whether they are instructed about and exercise their pelvic floor muscles.

## Materials and methods

### Design and participants

The present study was a secondary analysis of data that were collected as part of a prospective study of contributing factors that influence exercise involvement, attendance and dropout in a fitness club setting (*Fitness clubs — a venue for public health?* [17]. In the original study, participants were recruited between October 2015 and November 2017. The eligibility criteria were: < 4 weeks’ membership, untrained, ≥ 18 years old, literate in a Scandinavian language, healthy and not pregnant. Untrained was defined as exercising < 60 min once a week at moderate or vigorous intensity. Being healthy was defined as no severe disease or pathology. There were 676 new members from 25 multipurpose fitness clubs (resistance and cardio exercise rooms and group exercise classes) in Oslo, Norway, who expressed interest in participating in the study; however, 148 did not respond after the first email, and 278 did not meet the eligible criteria. Hence, 250 fitness club members [50% (*n* = 125) female] were included and followed for 1 year. More details on the research project are published elsewhere [17, 18].

### Ethical approval

All participants signed an informed consent form, following the Helsinki Declaration. The Regional Committee for Medical and Health Research Ethics, Southern Norway, Oslo, revised the project and complete data collection (REK 2015/1443 A) and concluded that the study did not require full review according to the act on medical and health research (Health Research Act 2008). The study was approved by the Norwegian Social Science Data Service (NSD 44135) and was financed by a PhD position (CG) at the Norwegian School of Sports Sciences (NSSS). No economic compensation was given to the participants.

### Outcome measures

A standardized, multidimensional, electronic questionnaire (SurveyXact, www.survey-xact.no) was answered at onset (52 questions) and after 3, 6 and 12 months (65 questions) of fitness club membership. The questionnaire took approximately 25 min to complete and was fully answered at the four time points by 125, 116, 107 and 91 women, respectively. A total of 90 answered at all time points. Up to three emails and one telephone reminder were sent to participants who did not respond. Losses to follow-up included life situation (*n* = 8), injury/disease (*n* = 4) and unknown reasons (*n* = 22).

#### Questionnaire

The questionnaire contained information about age, body weight and height, smoking, level of education, total household income, occupation, cohabitation and children. At 3, 6 and 12 months' follow-up, the participants also reported on exercise involvement in the last 4 weeks. The questions and response options were: (1) “Are you still a fitness club member?”: “yes” or “no.” (2) “Have you been exercising regularly?”: “yes” or “no.” (3) “Have you attended group exercise classes?”: “yes” or “no.” (4) “How often have you attended the following group exercise classes: aerobic dance/Zumba, boot-camp/CrossFit, biking/spin classes, strength training/BodyPump, yoga/Pilates”: “rarely or never,” “once a month,” “two to three times a month,” “once a week,” “two to three times a week,” “four to five times a week” or “six to seven times a week.”

If the participant responded “yes” to exercising regularly (question 2), their average number of sessions/week was obtained. With respect to self-reported exercise habits, the women were classified with either high or low exercise frequency. As recommended by Garber et al. [19], high exercise frequency was defined as visiting the fitness club ≥ two times a week in the last month and low exercise frequency as visiting the fitness club ≤ one time a week in the last month.

##### Data processing and questions about PFMT and UI

Awareness and knowledge about PFMT were assessed by a single question: “Do you know how to train your pelvic floor muscles?”: “yes,” “no” or “I do not know.” The women were also asked if PFMT had been part of their exercise routine during the last 4 weeks: “Do you train the pelvic floor muscles?”: “yes,” “no” or “I do not know,” and to report the average training frequency (the number of days they performed PFMT per week). As the most optimal dosage for effective PFMT is still not known, a training protocol following general strength-training principles is recommended [20]. Hence, similarly to exercise frequency, we divided the women into two PFMT groups (≥ two times a week in the last month and < two times a week or no PFMT in the last month).

Adherence to regular exercise and PFMT was defined as a minimum of two sessions/week throughout all follow-ups (at 3, 6 and 12 months). At 3, 6 and 12 months, the women were also asked if they had received any information or guidance about PFMT: “Have you received any coaching/supervision on pelvic floor muscle training by the fitness club staff/team?”: “yes” or “no.”

UI was measured using a modified version of the Subjective Health Complaints Inventory (SHC Inventory) and was assessed by the following questions: Have you experienced urinary leakage the past 4 weeks, and how bothered are you by the leakage?” The women were asked to evaluate this question on a symptom scale, with higher scores indicating “more” bothered by UI (0 = not at all to 3 = a great deal). To calculate the prevalence, responses were dichotomized into absent (score 0) or present (score ≥ 1) UI. The symptom scale was also used to categorize the severity of UI into three groups: slight, moderate or severe [21].

According to the International Consultation on Incontinence Questionnaire-Urinary Incontinence Short Form (ICIQ-UI SF), a question was also asked about when the leakage occurred. The women were able to select more than one response (multiple response question). Leakage during physical activity, coughing, sneezing and laughing were classified as stress urinary incontinence (SUI), whereas leakage with the urge to void was classified as urgency urinary incontinence (UUI) [3, 4]. The same questions about PFMT and UI were asked on all four follow-ups. We did not ask participants with UI about conservative treatment such as pessaries, tampons or medication.

### Statistical analysis

All statistics were conducted with SPSS software V. 24 for Windows. Not all participants answered every question, as such individual questions may have varying response rates.

Selected background and health variables between participants reporting UI or no UI at first assessment were compared using the chi-squared test for proportions and independent sample t-tests for means.

Changes in the proportions of participants reporting UI and severity of UI among the four time points (onset of membership, 3 months, 6 months and 12 months) were calculated using Cochran’s Q test.

No adjusted analyses were conducted as crude analysis showed no significant differences in adherence to regular exercise and PFMT (minimum two sessions/week) at any time point between women with and without UI at 12 months of fitness club membership.

Results are presented as numbers with percentages or means with standard deviation (SD) as well as group differences with 95% confidence intervals (CIs) and *p* values.

## Results

Mean age was 34.3 (± 10.0) years, and 38.4% reported being parous. Mean BMI (kg/m²) was 25.1 (± 4.9), and 38.4% were characterized as overweight or obese (BMI ≥ 25). General background characteristics of participants reporting absent or present UI at onset of fitness club membership are shown in Table 1. There were two differences between the groups: women with UI were older (9.2 years, 95% CI 4.7 to 13.7, *p* < 0.01), and a higher proportion were parous (30.2%, 95% CI 7.6 to 50.1, *p* < 0.01).

**Table 1:** General characteristics of the participants at onset of fitness club membership, divided into absent or present urinary incontinence (UI) (*n* = 125). Results are presented as mean (± SD) or number (and percentage)

Variable	No UI (*n* = 104)	Yes UI (*n* = 21)	*p* value
Age in years [mean (SD*)]	32.8 (9.5)	42.0 (8.9)	< 0.01
Age groups [mean (SD)]			< 0.01
< 40 years	80 (76.9)	10 (47.6)	
≥ 40 years	24 (23.1)	11 (52.4)	
BMI** (kg/m2) [mean (SD)]	24.9 (4.7)	26.0 (6.1)	0.34
BMI groups [*n* (%)]			0.65
< 25	65 (62.5)	12 (57.1)	
≥ 25	39 (37.5)	9 (42.9)	
Daily smoker*** [*n* (%)]	11 (10.6)	1 (4.7)	0.41
Children*** [*n* (%)]	28 (26.9)	12 (57.1)	< 0.01
Cohabitation/married*** [*n* (%)]	57 (54.8)	15 (71.4)	0.16
University/college education [*n* (%)]			0.75
No higher education	22 (21.2)	3 (14.4)	
< 4 years	39 (37.5)	7 (33.3)	
≥ 4 years	43 (41.3)	11 (52.4)	
Total household income [*n* (%)]			0.80
≤ 87,500 USD	65 (62.5)	11 (52.4)	
> 87,500 USD	39 (37.5)	10 (47.6)	
Occupation [*n* (%)]			
≥ 50%	80 (76.9)	15 (71.5)	0.60
Sick leave	4 (3.8)	1 (4.7)	0.84

*SD, standard deviation

**BMI, body mass index

***Answers to yes–no questions

### Prevalence of UI

Twenty-one women (16.8%) reported UI at the onset of fitness club membership. Of these, 15 (71.4%) perceived the UI to be slight, whereas 6 (28.5%) had moderate or severe UI. The majority reported SUI (Table 2).

**Table 2:** Prevalence, severity and type of urinary incontinence (UI) across four time points: at onset, 3, 6 and 12 months of fitness club membership. Results are presented as number (and percentage)

Variable	Onset (*n* = 125)	3 months (*n* = 116)	6 months (*n* = 107)	12 months (*n* = 91)	*p* value
UI					0.11
No	104 (82.2)	101 (87.1)	86 (80.4)	74 (81.3)	
Yes	21 (16.8)	16 (13.8)	21 (19.6)	17 (18.7)	
Severity of UI					
Slight	15 (71.4)	11 (73.3)	16 (76.2)	12 (70.6)	0.18
Moderate	4 (19.0)	2 (13.3)	5 (23.8)	4 (23.5)	–
Severe	2 (9.5)	2 (13.3)	0 (0)	1 (5.9)	–
Types of UI*					
Leaks with activity (SUI)	16 (76.2)	11 (73.3)	12 (57.1)	11 (64.7)	0.32
Leaks with coughing/sneezing (SUI)	16 (76.2)	16 (100.0)	13 (61.9)	10 (58.8)	0.11
Leaks with laughing (SUI)	5 (23.8)	4 (25.0)	3 (14.3)	4 (23.5)	–
Leaks before getting to toilet (UUI)	11 (52.4)	4 (25.0)	11 (52.4)	10 (58.8)	0.13

*Multiple response questions (tick all that apply)

Repeated measures of UI, collected from each participant across the four time points (onset of membership, 3 months, 6 months and 12 months), showed no changes in the proportions or severity of UI (Table 2).

### Frequency of exercise and performing PFMT

At 3, 6 and 12 months, the proportions of women reporting regular exercise (minimum two sessions/week) were 59%, 57% and 46%, respectively (*p* = 0.062) (Table 3). Less than 1/3 (30%) reported adherence to regular exercise at all time points.

**Table 3:** Proportions reporting performing regular exercise and PFMT across four time points: at onset, 3, 6 and 12 months of fitness club membership. Results are presented as number (and percentage) or mean (± SD)

Variable	Onset (n = 125)	3 months (n = 116)	6 months (n = 107)	12 months (n = 91)	*p* value
Regular exercise* [*n* (%)]					0.06
Yes	–	68 (58.6)	61 (57.0)	42 (46.2)	
No	–	48 (41.4)	46 (43.0)	49 (53.8)	
Awareness of PFMT [*n* (%)]	83 (66.4)	88 (75.9)	80 (74.8)	70 (76.9)	0.01
Coaching/supervision on PFMT [*n* (%)]	–	11 (9.5)	7 (6.5)	6 (6.6)	0.69
Regular PFMT* [*n* (%)]					0.11
Yes	21 (16.8)	31 (26.7)	29 (27.1)	20 (22.0)	
No	104 (83.2)	85 (73.3)	78 (72.9)	71 (78.0)	
PFM/week** [Mean (SD)]	0.46 (1.6)	1.2 (2.2)	3.2 (3.7)	2.4 (3.3)	< 0.01

*Regular exercise and PFMT were both defined as a minimum of two sessions/week

**Number of days doing PFMT per week

There was an increase in women reporting awareness/knowledge of PFMT at follow-up measurements (*p* = 0.01), yet repeated measures showed no increase in the proportion of women performing regular PFMT (minimum two sessions/week) throughout the first year of fitness club membership (Table 3). Among those reporting PFMT, there was an increase in the number of days they performed PFMT per week (*p* < 0.01) (Table 3). About 1/5 (22.2%) reported performing PFMT at least twice weekly at all follow-ups. Less than 8% had received information about PFMT by the fitness club staff.

Adherence to regular exercise and PFMT at all follow-up points (at 3, 6 and 12 months) did not show any associations with absent or present urinary UI at 12 months (Table 4). Of those women who reported UI at 12 months, 14 of 17 (82.4%) did not perform regular PFMT (Table 4).

**Table 4:** Comparison of adherence* to exercise and pelvic floor muscle training (PFMT) between participants reporting absent or present urinary incontinence (UI) at 12 months of fitness club membership (*n* = 90). Results are presented as number (and percentage)

Variable	No UI (*n* = 73)	Yes UI (*n* = 17)	*p* value
Regular exercise			0.49
Yes	23 (31.5)	4 (23.5)	
No	50 (68.5)	13 (76.5)	
Regular PFMT			0.63
Yes	17 (23.3)	3 (17.6)	
No	56 (76.7)	14 (82.4)	

*Adherence to regular exercise and PFMT was defined as a minimum two sessions/week throughout all follow-up points (at 3, 6 and 12 months)

Descriptive data of high- and low-impact group exercise classes ≥ once weekly, divided into absent or present UI, are summarized in Table 5. At 3, 6 and 12 months, 1, 1 and 0 with leakage, compared with 8, 9 and 5 without leakage reported high-impact group exercises such as aerobic dance/Zumba and boot camp/CrossFit, respectively.

**Table 5:** Types of group exercise classes ≥ once weekly (divided into high and low impact) and absent or present urinary incontinence (UI) at 3, 6 and 12 months of fitness club membership. Results are presented as number (and percentage)

Types of group class	months (*n* = 116)		6 months (*n* = 107)		12 months (n = 91)	
	No UI (*n* = 101)	Yes UI (*n* = 16)	No UI (*n* = 86)	Yes UI (n = 21)	No UI (*n* = 74)	Yes UI (n = 17)
High impact	**8 (7.9)**	**1 (6.3)**	**9 (10.5)**	**1 (4.8)**	**4 (5.4)**	**0 (0)**
Aerobic dance/Zumba	6	1	5	1	4	0
Boot camp/CrossFit	2	0	4	0	0	0
Low impact	**30 (29.7)**	**12 (75.0)**	**19 (22.1)**	**7 (33.3)**	**14 (18.9)**	**2 (11.8)**
Biking (spin classes)	12	5	7	3	7	0
Strength training (Body Pump)	10	4	11	2	4	2
Yoga/Pilates	8	3	1	2	3	0

## Discussion

In the present study, no changes were observed in proportions or severity of UI across the first year of fitness club membership. Most women perceived the symptoms to be slight, and the prevalence of UI was lower than has been reported in the general female population [2] as well as other studies of women participating in different sports and exercise classes [8, 15, 16], including a study of young nulliparous women (20–25 years) attending gyms [14] and group fitness instructors [7, 13].

We purposely aimed to recruit a typical sample of beginner exercisers at fitness clubs, and our participants are therefore more diverse than those of previous studies with respect to age, BMI, and parous and nulliparous women [8, 22]. Mean age was about 35 years and nearly 40% had a BMI ≥ 25 and as such were classified as overweight or obese. Because around one third of the study sample was parous, we expected to find a higher prevalence of UI than we observed. We did, however, find that women aged ≥ 40 years had the highest prevalence of UI (52.4%), in accordance with the literature [5, 8].

Unfortunately, the available studies on the epidemiology of female UI have used different definitions of UI, and the populations studied are heterogeneous. We investigated UI as a part of 16 common health problems based on a modified Subjective Health Complaints Inventory (SHC Inventory), simply scoring UI by how the complaint was perceived and reported by the participants in the past 4 weeks, without referring to any diagnostic categories. In addition, one item from the ICIQ-UI SF about when the leakage occurred was added. For future studies, we would advise the sole use of the ICIQ-UI SF [3], which is a brief and robust questionnaire, allowing the assessment of UI frequency, severity and impact on quality of life (QoL). ICIQ-UI SF can be used for clinical research for free; however, permission is required. On the other hand, the time frame of the past 4 weeks used in the present study is in line with ICIQ-UI SF [3]. Also, how we dichotomized the responses into absent (score 0) or present (score 1 and higher) UI was as recommended in the last International Urogynecological Association/International Continence Society joint report on the terminology for Female Pelvic Floor Dysfunction, where UI was defined as the complaint of any type of involuntary loss of urine [1].

Even though controversies exist on the role of physical activity/exercise on UI [9], and several studies have reported that incontinence during exercise is common, the impact of UI on exercise adherence varies in different studies [8]. Some cross-sectional studies have found that UI may be a barrier to physical activity and that incontinent women modify and cease their exercise behavior because of fear of leakage and odor [8, 10]. On the other hand, a study from Finland reported that becoming continent (after successful conservative or surgical treatment) did not result in increased physical activity [23]. In the present study, there was no association between UI and exercise, and at 12-month follow-up, women with UI were not more likely to report low levels of exercise (≤ one time a week or no exercise). We observed, however, that at onset of fitness club membership, very few participants (6/125) reported moderate or severe UI. This may imply that a higher symptom score of UI was a barrier for exercise or that women being “more” bothered by UI may have selected other arenas for physical activity or stayed inactive. In addition, 22 participants were lost to follow-up at 12 months, without providing an explanation for this. Hence, we do not know if UI was a reason for non-participation.

PFMT is recommended as the first-line treatment option for UI [12]. Yet, in our study, there was no difference in the number with absent or present leakage and report of PFMT. Some studies have described that women were motivated to carry out PFMT if they had a current problem with UI, and especially severity of UI was associated with motivation for treatment [24]. In the present study, most women perceived their UI to be slight. This could be an explanation for the large gap between awareness/knowledge of PFMT (about 75%), and performing PFMT (about 25%) regularly at 3, 6 and 12 months of follow-up.

UI can interfere with daily life activities and lead to decreased quality of life if left untreated. It is important to highlight that PFMT is also a key component for prevention of UI [8]. Therefore, in light of this and that UI affects exercisers at higher rates than inactive women [8, 22], PFMT should probably be incorporated into popular group exercise classes and training programs for women at fitness clubs. Also, with respect to the social stigma around UI, and because many women are hesitant to talk about their symptoms because of embarrassment [22], a more population-based strategy for inclusion of PFMT would undoubtedly be helpful.

In a literature overview, Bø and Sherburn [25] reported that > 30% of women do not contract their PFM correctly at first consultation, whereas a more recent study found that most women are able to correctly contract their PFM after a simple verbal instruction [26]. Hence, data are not conclusive. A recent study from Scotland reported that there was widespread willingness by instructors to incorporate PFMT into fitness classes if given appropriate training [13].

Few women (7.5%) had received coaching or supervision in PFMT by the fitness club staff members, which is in line with what fitness instructors self-report [13]. We believe there is huge potential for improvement in PFMT guidance in exercise classes and at fitness clubs, along with the promotion of pelvic floor friendly exercise options. A systematic review [27] has also concluded that common exercise classes taught in fitness clubs, such as Pilates and yoga, have not been proven effective in elevating the bladder neck and might even result in bladder neck descent, further weakening of the pelvic floor. However, there is obviously a need for PFMT to be taught properly to avoid adverse effects from incorrect practices.

### What is known and what this study adds

To date, there are about 90 million female fitness club members worldwide, representing one of the world’s biggest activity settings for women (The IHRSA Global Report 2019). We also know that incontinence during exercise (SUI) is a common problem and that a quarter of all women will experience UI at some stage in their life [2]. Cure rates of PFMT vary between 35%–80%, depending on the amount of supervision and training, and the training has no known side effects [28]. Educating and assisting fitness instructors in how to correctly teach PFMT could be a good method for preventing and treating UI, which would lower the economic burden and need for other medical and health care services (Continence Foundation of Australia, www.continence.org.au).

In 2004, Bø [9] described two contradictory hypotheses on the effect of general fitness training on the pelvic floor. The first claims that regular exercise makes the pelvic floor muscles co-contract, resulting in stronger muscles, and thus preventing UI. The second claims that repetitive increases in intra-abdominal pressure and ground reaction forces, combined with sudden changes in pressure during exercise, could overload and fatigue the pelvic floor muscles, which may increase the risk of leaking. In cross-sectional study designs, it is difficult to establish whether habitual exercise increases or decreases the risk of UI, because women with UI during exercise may avoid being active. Hence, prospective studies are needed to begin to understand causality [8]. We did not find increased or decreased prevalence of UI among previously untrained women starting a regular exercise regimen the first year of fitness club membership.

### Strengths and limitations

To our knowledge, this is the first study of UI in a group of beginner fitness club members, all considered untrained by inclusion. In addition, we used a prospective observational design allowing us to study causality of exercise and UI. The participants were an age-diverse group of women (18–62 years), and the response rate was high, with 72% answering the questionnaire at all four time points. The use of an electronic questionnaire to gather responses quickly and eliminate the costs associated with printing and distributing paper-based questionnaires may also be considered a strength. We recruited from 25 fitness clubs and consider the participants representative of an untrained population, because they showed a low level of fitness as measured by VO_2max_ at onset of fitness club membership [18].

A potential limitation is that data from nulliparous and parous participants were analyzed together. By treating the women as one group, we lost information: just by analyzing the proportions, we saw that UI was much higher among parous women. The reason for the above grouping was that few reported UI (*n* = 21), so there was a lack of statistical power to do sub-group analyses. Our sample size was also too small to analyze changes in severity of UI (moderate and severe) and types of UI across the first year of membership. Finally, self-reported data are subject to under- or over-reporting, depending on the question asked and the social desirability of the response given [29].

## Conclusion

In an age diverse group of new beginner exercisers, about 17% reported UI at onset of fitness club membership, with no changes in prevalence or severity of UI across the first year. This study did not find a higher prevalence of UI in women who reported regular exercise (minimum two sessions/week) compared with those who did not. Less than 8% reported having received guidance on PFMT by the fitness club staff. One out of five reported doing PFMT at least twice weekly at all follow-ups; however, we did not observe an association between this measure of PFMT adherence and continence.
